# The neuroprotective mechanism of cinnamaldehyde against amyloid-β in neuronal SHSY5Y cell line: The role of N-methyl-D-aspartate, ryanodine, and adenosine receptors and glycogen synthase kinase-3β

**Published:** 2019

**Authors:** Masoumeh Emamghoreishi, Majid Reza Farrokhi, Atena Amiri, Mojtaba Keshavarz

**Affiliations:** 1 *Department of Pharmacology, School of Medicine, Shiraz University of Medical Sciences, Shiraz, Iran.*; 2 *Department of Neuroscience, School of Advanced Medical Sciences and Technologies, Shiraz University of Medical Sciences, Shiraz, Iran.*; 3 *Shiraz Neuroscience Research Center, Shiraz University of Medical Sciences, Shiraz, Iran.*

**Keywords:** Adenosine, Cinnamaldehyde, Dantrolene, Glycogen synthase kinase, Neuroprotection, N-methyl-D-aspartate

## Abstract

**Objective:**

Cinnamaldehyde may be responsible for some health benefits of cinnamon such as its neuroprotective effects. We aimed to investigate the cinnamaldehyde neuroprotective effects against amyloid beta (Aβ) in neuronal SHSY5Y cells and evaluate the contribution of N-methyl-D-aspartate (NMDA), ryanodine, and adenosine receptors and glycogen synthase kinase (GSK)-3β, to its neuroprotective effects.

**Materials and Methods:**

After seeding the cells in 96-well plates, adenosine (20, 40, 80, and 120 µM), NMDA (20, 40, 80, and 120 µM), and dantrolene (as a ryanodine receptor antagonist; 2, 4, 6, 8, and 16 µM) were added to the medium containing Aβ25-35 and/or cinnamaldehyde. The 3-[4,5-dimethylthiazol-2-yl]-2,5-diphenyl tetrazolium bromide method was used to assess neurotoxicity and western blot to measure the GSK-3β protein level.

**Results:**

Cinnamaldehyde (15, 20, 23, and 25 μM) significantly reversed Aβ-induced toxicity in SHSY5Y neuronal cells. Adenosine (20, 40, 80 and 120 μM) inhibited the neuroprotective effects of cinnamaldehyde (15 μM). NMDA (20, 40, 80, and 120 μM) reduced cinnamaldehyde (15 and 23 μM) neuroprotective effects against Aβ neurotoxicity. Dantrolene (2, 4, 8, and 16 μM) significantly reduced cinnamaldehyde (15 μM) neuroprotective effects. Cinnamaldehyde (15 and 23 μM) suppressed the Aβ-induced increment of GSK-3β protein level.

**Conclusion:**

NMDA and adenosine receptors suppression together with ryanodine receptors stimulation may be relevant to cinnamaldehyde neuroprotective effects against Aβ neurotoxicity. Moreover, the inhibition of GSK-3β may contribute to the cinnamaldehyde neuroprotection.

## Introduction

Alzheimer's disease (AD) is the most popular neurodegenerative disorder which is characterized by cognitive impairment especially in older persons (Lindeboom and Weinstein, 2004 ▶). The extracellular deposition of β-amyloid (Aβ) plaques and aggregation of hyperphosphorylated form of tau protein (neurofibrillary tangles) are classic signs of AD (Checler, 1995 ▶; Delacourte and Buée, 2000 ▶). Recent evidence has shown that glycogen synthase kinase-3β (GSK-3β) may enhance the Aβ-induced tau phosphorylation and progression of AD pathophysiology (Hernandez et al., 2013 ▶). Furthermore, deregulated calcium homeostasis may activate apoptotic pathways and cause neural cell death in AD (LaFerla, 2002 ▶). Abnormal intracellular calcium homeostasis may precede pathophysiological changes in AD (Chui et al., 1999 ▶).

Calcium as an essential second messenger (Berridge et al., 1998 ▶) regulates neural cell function and viability (Berridge et al., 1998 ▶). N-methyl-D-aspartate (NMDA), adenosine, and ryanodine receptors are important systems that regulate the intracellular calcium concentration (Gomes et al., 2011 ▶; Lüscher and Malenka, 2012 ▶; McPhersonx et al., 1991 ▶). These receptors may connected to the pathophysiology and treatment of AD (Bruno et al., 2012 ▶; Danysz and Parsons, 2012 ▶). 

*Cinnamomum verum* extract showed a neuroprotective effect in AD models (Frydman-Marom et al., 2011 ▶; Peterson et al., 2009 ▶). Moreover, the bioactive compounds of cinnamon were shown to reduce GSK-3β activity in the peripheral tissues (Imparl-Radosevich et al., 1998 ▶). The therapeutic potential of cinnamon is mainly related to its phytochemicals like cinnamaldehyde, cinnamyl, and eugenol (Stavinoha and Vattem, 2015 ▶). However, the active compound responsible for the neuroprotective effects of cinnamon is unknown. 

Cinnamaldehyde is one of the main components of cinnamon (Chen et al., 2016 ▶) with beneficial effects on inflammatory and oxidative stress, blood glucose and malignancy (Anderson et al., 2004 ▶; Ataie et al., 2019 ▶; Kwon et al., 1997 ▶; Zhao et al., 2015 ▶). Cinnamaldehyde may be also responsible for some health benefits of cinnamon such as its neuroprotective effects (Stavinoha et al., 2015 ▶). In this regard, cinnamaldehyde was shown to reduce neuroinflammation, microglia activation and tau aggregation (Ho et al., 2013 ▶; Peterson et al., 2009 ▶). However, the potential effects and mechanism of action of cinnamaldehyde against Aβ neurotoxicity are relatively unknown. Therefore, we aimed to assess the cinnamaldehyde neuroprotective effects in SHSY5Y cells exposed to Aβ and the involvement of NMDA, ryanodine and adenosine receptors in its neuroprotective effects. Moreover, we tried to explore the relationship between GSK-3β inhibition and neuroprotective effects of cinnamaldehyde. 

## Materials and Methods

Human SHSY5Y neuroblastoma cell line were obtained from Pasteur Institute (Iran). The cell culture reagents including DMEM/F12, fetal bovine serum (FBS), and penicillin-streptomycin were purchased from Gibco^® ^life technologies^™ ^(USA). We purchased Aβ25-35, NMDA, cinnamaldehyde (W228613), dantrolene sodium salt, and adenosine from Sigma-Aldrich (USA). The anti-actin, anti-GSK-3β, and anti-phosphorylated (p)-GSK-3β antibodies were obtained from Cell Signaling Technology^®^ (USA). the phosphate buffered solutions (PBS) was used as the solvent of cinnamaldehyde, dantrolene, adenosine, and NMDA.

**Neuronal Cell Culture**

The seeded cells (1×10^5 ^cells/well) were kept in 96-well plates and in a Dulbecco's modified Eagle's medium (DMEM) plus Ham's nutrient mixture F-12 (1:1). Other reagents including 10% FBS, 100 U/ml penicillin, and 100 µg/ml streptomycin were added to the plates. Then, the plates were maintained in the condition of 95% air/5% CO_2_ at 37^◦^C. 

**Treatment**

We incubated Aβ25–35 for four days at 37°C to induce the aggregation process. The best concentrations of Aβ25-35 and cinnamaldehyde were determined in a pilot study. In the pilot study, we used different concentrations of cinnamaldehyde (1-50 μM) and chose the concentrations of 15 and 23 µM. We also chose 20 µM as the best concentration of Aβ23-35. After replacement of serum-free media, Aβ23-35 (20 µM), cinnamaldehyde (15 and 23 µM) or both was added to the plates. Adenosine (20, 40, 80, and 120 µM), NMDA (20, 40, 80, and 120 µM), and dantrolene (2, 4, 6, 8, and 16 µM) were added to the culture media containing Aβ23-35, cinnamaldehyde or both in independent experiments (4 experiment).

**Cell viability assay**

Cell viability was measured using 3-[4,5-dimethylthiazol-2-yl]-2,5-diphenyl tetrazolium bromide (MTT) reagent (5 mg/ml) 24h after the treatment. The cells were incubated with MTT for 4h, and then, the precipitate was dissolved in 100 µl of dimethyl sulfoxide (DMSO). The absorbance was determined at a 570 nm by a microplate reader (Synergy HT, Biotek®). 

**Total protein determination**

The SHSY5Y cells were transported to the 6-well plates (10^6^ cells/ml) for protein analysis. After incubation with cinnamaldehyde and Aβ, cells were removed by centrifuging at 14000 g for 5 min. The radio-immunoprecipitation assay lysis (RIPA) buffer containing protease and phosphatase inhibitor cocktail was used to lyse the cells. The lysate cells were centrifuged at 19000 g for 25 min at 4^◦^C to eliminate cell debris. The soluble part was used to determine total protein level. The Lowry method was used to determine the total protein level (Lowry et al., 1951).

**Western blot analysis**

SDS-PAGE separated the proteins and equal amounts of proteins were transblotted onto polyvinylidene fluoride (PVDF) membrane. For blocking, the membrane was incubated with 5% bovine serum albumin (BSA) for 1 hr at room temperature. Then, the membrane incubated with the GSK-3β rabbit monoclonal antibody, p-GSK-3β (Ser9) antibody, and actin overnight at 4^◦^C. The membrane was washed with TBST (tris-buffered saline and tween 20) buffer. After that, the anti-rabbit IgG, HRP-linked antibody (7074s, Cell Signaling) (1:25000) was poured on the membrane and kept for 1 hr at 37^◦^C. Then, we scanned the membrane using an enhanced chemiluminescence kit (GE Healthcare) and ChemiDoc™ XRS+ imaging System. The bands were analyzed by image-J software. 

**Statistical analysis**

The Shapiro-Wilk test was used for the evaluation of the normal distribution. The data were analyzed with the one-way analysis of variance (ANOVA) followed by the Bonferroni test. The significance level was set at 0.05. All the analyses were executed using SPSS software version 23. 

## Results

**Cinnamaldehyde neuroprotection against Aβ neurotoxicity**

**Figure 1:**
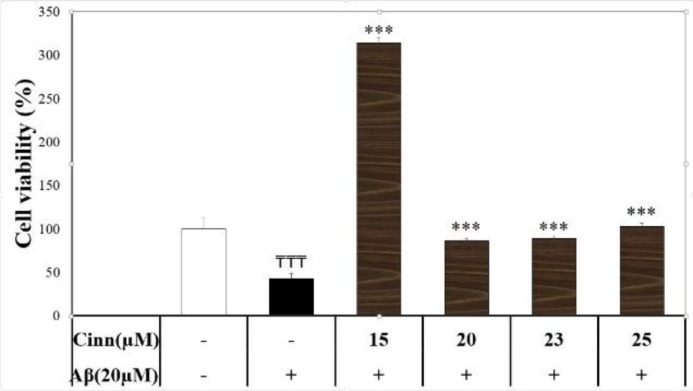
Effect of cinnamaldehyde (Cinn) on the viability of SHSY-5Y cells in the presence of beta amyloid (Aβ). The percent of viable cells was determined using MTT assay and normalized against untreated cells. Data are presented as the mean ± standard error of the mean of four experiments. ₸₸₸: p<0.001 compared to the vehicle-treated cells (white column); ***: p<0.001 compared to the Aβ-treated cells (black column)

Aβ (20 μM) reduced neural cell survival by 57%. Cinnamaldehyde (15, 20, 23, and 25 μM) significantly reversed Aβ neurotoxicity (F(5)=14.66, p=0.000) (Figure 1). However, cell viability was not different between cinnamaldehyde 15 μM and cinnamaldehyde 23 μM groups (p>0.05).

**Adenosine interference with cinnamaldehyde neuroprotection against Aβ**

**Figure 2:**
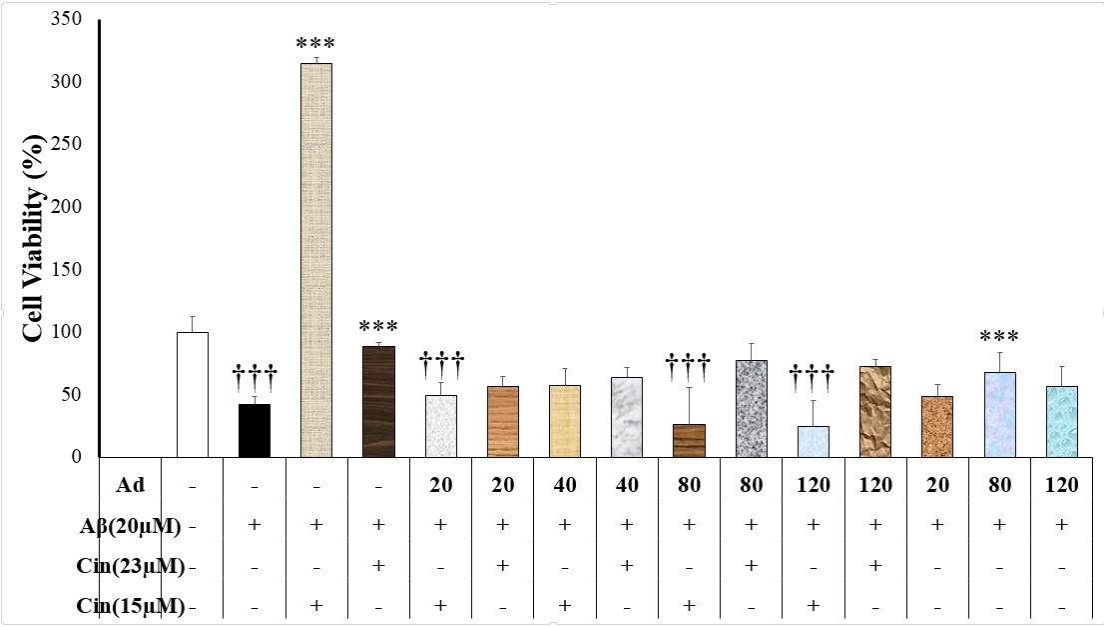
Effect of cinnamaldehyde (Cinn) on the viability of SHSY-5Y cells in the presence of adenosine (Ad) and beta amyloid (Aβ). The percent of viable cells was determined using MTT assay and normalized against untreated cells. Data are presented as the mean ± standard error of the mean of four experiments; ***: p<0.001 compared to the Aβ-treated cells (black column); †††: p<0.001 compared to the Aβ+Cinn (15 μM)-treated

Adenosine (without cinnamaldehyde) only at the concentration of 80 μM produced a neuroprotective effect against Aβ neurotoxicity (p=0.043) (Figure 2). Adenosine (20, 40, 80 and 120 μM) inhibited cinnamaldehyde neuroprotective effects (15 μM) against Aβ (F(9)=10.36, p=0.000) (Figure 2). Adenosine had no effect on cinnamaldehyde (23 μM) neuroprotective effects (Figure 2). 

**NMDA interference with cinnamaldehyde neuroprotection against Aβ neurotoxicity**

**Figure 3:**
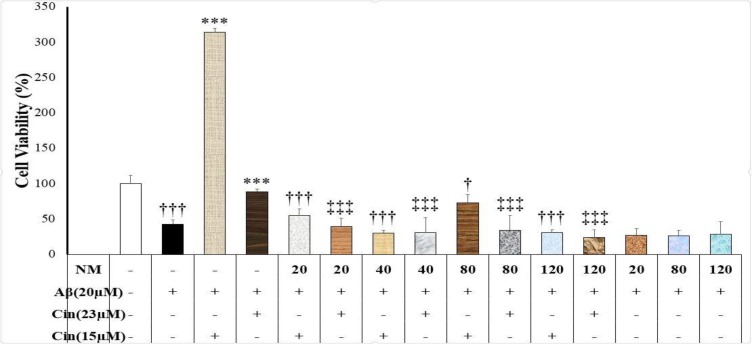
Effect of cinnamaldehyde (Cinn) on the viability of SHSY-5Y cells in the presence of N-Methyl-D-aspartate (NMDA) and beta amyloid (Aβ). The percent of viable cells was determined using MTT assay and normalized against untreated cells. Data are presented as the mean ± standard error of the mean of four experiments. ***: p<0.001 compared to the Aβ-treated cells (white column); †: p<0.05 and †††: p<0.001 compared to the Aβ+Cinn (15 μM)-treated cells; ‡‡‡: p<0.001 compared to the Aβ+Cinn (23 μM)-treated cells

The present study showed that NMDA (20, 40, 80, and 120 μM) decreased the cinnamaldehyde (15 and 23 μM) neuroprotective effects (F(9)=26.41, p=0.000, and F(9)=14.35, p=0.000, respectively) (Figure 3). Moreover, NMDA alone (20, 80, and 120 μM) had no effect on Aβ neurotoxicity (F(3)=0.66, p=0.59). 

**Dantrolene interference with the cinnamaldehyde neuroprotection against Aβ neurotoxicity**

**Figure 4:**
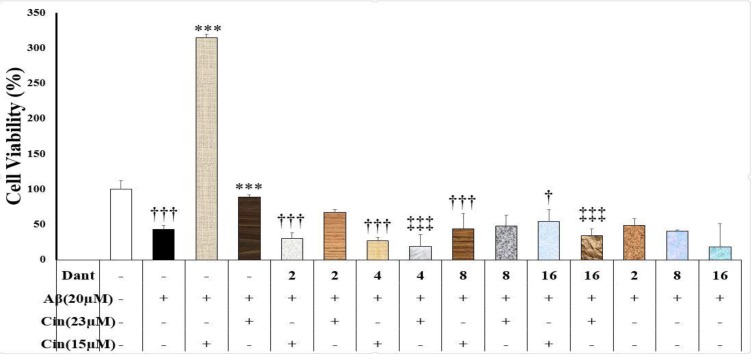
Effect of cinnamaldehyde (Cinn) on the viability of SHSY-5Y cells in the presence of dantrolene (Dant) and beta amyloid (Aβ). The percent of viable cells was determined using MTT assay and normalized against untreated cells. Data are presented as the mean ± standard error of the mean of four experiments. ***: p<0.001 compared to the Aβ-treated cells (white column); †: p<0.05 and †††: p<0.001 compared to the Aβ+Cinn (15 μM)-treated cells; ‡‡‡: p<0.001 compared to the Aβ+Cinn (23 μM)-treated cells

Dantrolene (2, 4, 8, and 16 μM) significantly reduced cinnamaldehyde (15 μM) neuroprotective effects (F(9)=13.76, p=0.000) (Figure 4). 

Moreover, dantrolene (4 and 16 μM) suppresed the neuroprotective effects of cinnamaldehyde (23 μM) (Figure 4). Furthermore, dantrolene alone (2, 8 and 16 μM) had no effect on the Aβ-induced neurotoxicity (p>0.05). 

**Cinnamaldehyde effects on the GSK-3β protein**

**Figure 5:**
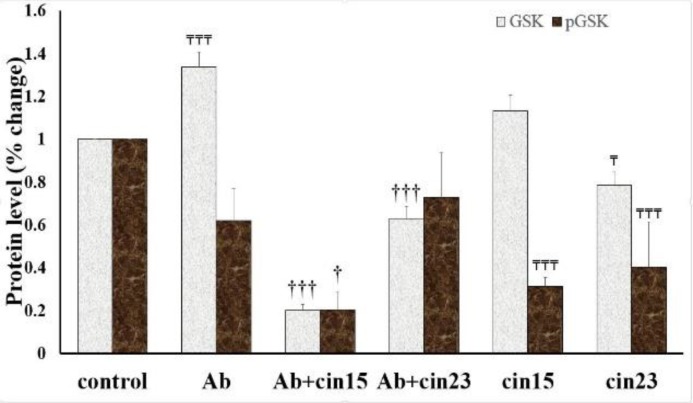
Effect of cinnamaldehyde (Cin) on the level of total and phosphorylated (p)-glycogen synthase kinase (GSK)-3β in SHSY-5Y cells in the presence of beta amyloid (Aβ). The protein level was determined using Western blot analysis and normalized against untreated control cells. Data are presented as the mean ± standard error of the mean of four experiments. †: p<0.05 and †††: p<0.001 compared to the Aβ-treated cells; ₸: p<0.05 and ₸₸₸: p<0.001 compared to the vehicle-treated cells (control)

The expression of GSK-3β protein was different in the studied groups (F(5)=172.483, p=0.000) (Figure 5). Treatment of neuronal cells with Aβ increased the total GSK-3β protein levels compared to the control group (p=0.000) (Figure 5). Cinnamaldehyde 15 and 23 µM diminished the Aβ effects on the total GSK-3β protein levels (p<0.001) (Figure 5). Cinnamaldehyde (23 µM) decreased the total GSK-3β protein compared to the control group (p=0.004) (Figure 5). 

The p-GSK-3β protein levels in the studied groups were different (F(5)=12.972, p=0.000) (Figure 5). Aβ decreased, though with no statistical significance, the p-GSK-3β protein level (p=0.057) (Figure 5). The p-GSK-3β protein level in the Aβ+cinnamaldehyde (15 µM) group was lower than the Aβ-treated group (p=0.034) (Figure 5). However, the p-GSK-3β protein level in the Aβ+cinnamaldehyde (23 µM) group was not different from the Aβ group (p=0.934) (Figure 5). Moreover, the p-GSK-3β protein level in the cinnamaldehyde (15 and 23 µM) groups were lower compared to the control group (p=0.001 and p=0.003, respectively) (Figure 5).

## Discussion

Our study showed that cinnamaldehyde, the main component of cinnamon, protected neuronal cells against Aβ neurotoxicity. Cinnamon extract inhibited Aβ neurotoxicity and improved cognitive function in an animal model of AD (Frydman-Marom et al., 2011 ▶). Furthermore, cinnamon decreased Aβ oligomerization and tau aggregation in cell culture and in an animal model of AD (Frydman-Marom et al., 2011 ▶; Peterson et al., 2009 ▶). Therefore, cinnamaldehyde may be regarded as an alternative therapy for the treatment of AD. 

The present study showed that cinnamaldehyde reduced GSK-3β protein level and prevented the Aβ-induced increases in GSK-3β. Similarly, it was shown that cinnamon extract suppressed the GSK activation and reduced tau phosphorylation in the transfected HEK293 cells (Donley et al., 2016 ▶). Moreover, cinnamon decreased the GSK-3β mRNA and protein levels in the liver and muscle of rats (Couturier et al., 2011 ▶). On the other hand, cinnamaldehyde did not affect GSK phosphorylation in primary astrocyte culture (Sartorius et al., 2014 ▶). Thus, our study revealed the inhibitory effects of cinnamaldehyde on the GSK-3β protein in a neuronal cell line and showed the contribution of this effect to the neuroprotection against Aβ-induced neurotoxicity. 

Adenosine is an important neuromodulator with potential roles in AD (Dall'Igna et al., 2007 ▶). However, the exact function of this endogenous substance should be clarified (Gomes et al., 2011 ▶). Our study showed that the activation of adenosine receptors might induce neuroprotection, while adenosine diminished the neuroprotective effects of cinnamaldehyde. These effects may imply that adenosine and cinnamaldehyde affect similar receptors and compete for binding to these receptors. To the best of our knowledge, there is no other study about possible interactions between cinnamaldehyde and adenosine receptors. However, other adenosine antagonist like caffeine protected neuronal cells against Aβ (Dall'lgna et al., 2003 ▶; Keshavarz et al., 2017 ▶). Moreover, an A2A agonist blocked the caffeine neuroprotective effects against Aβ (Dall'lgna et al., 2003 ▶). Caffeine and an A2A antagonist reversed Aβ-induced cognitive impairments in animal models (Dall'Igna et al., 2007 ▶). Thus, the adenosine receptors may be potential targets for the cinnamaldehyde neuroprotective effects. However, cinnamaldehyde only at a lower concentration diminished the effects of adenosine.

The inhibition of NMDA receptors may be involved the neuroprotective effects of cinnamaldehyde. Similarly, cinnamaldehyde protected PC12 cells against NMDA-induced neurotoxicity (Lv et al., 2017 ▶). Furthermore, cinnamon extract produced neuroprotection against glutamate in primary neuronal culture (Shimada et al., 2000 ▶) and protected neurons against glutamate and NMDA neurotoxicity in the primary chick embryo neuronal culture (Gomada et al., 2012 ▶). An animal study showed that cinnamaldehyde increased the glutamate release (Klafke et al., 2012 ▶). However, cinnamaldehyde produced no effect on the glutamate binding (Klafke et al., 2012 ▶). The discrepancies among the studies may arise from methodological differences or the nature of used neurons. 

Cinnamon neuroprotective effect may result from the modulation of intracellular calcium. Ryanodine receptors are sarcoplasmic reticulum calcium channels that modulate intracellular calcium in the brain and peripheral tissues (McPhersonx et al., 1991 ▶). Therefore, blockade of ryanodine receptors may diminish the neuroprotective effects of cinnamaldehyde. There are some controversies about the roles of ryanodine receptor in neurotoxicity and neuroprotection. In accordance with our study, dantrolene deteriorated Aβ-induced hippocampal neuronal damage in an animal model of AD (Zhang et al., 2010 ▶). Furthermore, the inability to up-regulate ryanodine receptor-3 may be the cause of neuronal vulnerability against multiple insults like Aβ, oxidative stress, and excitotoxicity (Allan Butterfield, 2002 ▶). Accordingly, activation of the ryanodine receptor may be a cellular mechanism to protect neurons in the initial stage of AD (Supnet and Bezprozvanny, 2010 ▶). On the other hand, dantrolene ameliorated cognitive dysfunction by decreasing Aβ load in an animal model of AD (Peng et al., 2012 ▶). 

GSK is a regulatory enzyme involved in several CNS functions like neuronal development and neurodegeneration (Bhat et al., 2004 ▶). Accordingly, GSK is an important target in the pathophysiology and treatment of neurodegenerative disorders including AD (P. Chen et al., 2007). The GSK-3β protein may be a mediator in the neurotoxic effects of Aβ (Phiel et al., 2003 ▶; Takashima, 2006 ▶). Our study showed that cinnamaldehyde reversed the Aβ effects on the GSK-3β protein levels. Similarly, Imparl-Radosevich and colleagues showed that cinnamon inhibited the GSK-3β activity in the peripheral tissue (Imparl-Radosevich et al., 1998 ▶). Thus, cinnamaldehyde neuroprotective effects may be due to GSK-3β inhibition. There is limited information regarding the cinnamaldehyde interactions with the GSK-3β protein. Our study showed that NMDA activation suppressed the neuroprotective effects of cinnamaldehyde. On the other hand, it was shown that NMDA stimulation enhance the activity of GSK-3β in primary neuronal culture and animal brain (Szatmari et al., 2005 ▶). Similarly, GSK-3β activation enhanced NMDA activity in neurons (Szatmari et al., 2005 ▶). The interaction between NMDA receptors and GSk-3β protein may induce neuronal toxicity. Inhibition of NMDA receptors and GSK-3 protein by cinnamaldehyde may produce neuroprotective effects. 

The application of non-selective agonist and antagonists of adenosine and ryanodine receptors may be the main limitation of this study. We assessed cinnamaldehyde effects on these two receptors. Future studies should employ selective modulators of these receptors. Moreover, the MTT method has some limitations. We suggest reassessing the results of this study by other apoptosis evaluation techniques. 

Cinnamaldehyde protected neurons against Aβ. The exact mechanism of cinnamaldehyde neuroprotective effects should be studied in future. However, the neuroprotective effects of cinnamaldehyde may be related to inhibition of NMDA and adenosine receptors together with stimulation of ryanodine receptors. Inhibition of the GSK-3β protein may enhance cinnamaldehyde neuroprotective effects.
